# Sweat Rate, Sweat Sodium Losses, and Body Composition in Professional Male Soccer Players in Southwest Colombia

**DOI:** 10.3390/medicina60010113

**Published:** 2024-01-07

**Authors:** Milton Fabian Suarez-Ortegón, Maria del Pilar Zea-León, Angelica Maria Astudillo-Gironza, Silverio Garzón, Gustavo Fabian Portela, Oscar Daniel Villarreal-Nieto

**Affiliations:** 1 Departamento de Alimentación y Nutrición, Facultad de Ciencias de La Salud, Pontificia Universidad Javeriana Seccional Cali, Cali 760021, Colombia; maria.zea@javerianacali.edu.co; 2 Asociación Deportivo Cali, Cali 760021, Colombiamedicoep@deportivocali.com.co (G.F.P.); 3 Departamento de Ciencias Básicas, Facultad de Ciencias de La Salud, Pontificia Universidad Javeriana Seccional Cali, Cali 760021, Colombia; silveriog@javerianacali.edu.co

**Keywords:** sweat rate, sweat sodium, fluid balance, muscle mass, fat mass, body mass

## Abstract

*Background and Objective*: Dehydration and hyperhydration impact athletes’ performance. Exploring the fluid balance concerning body composition might help estimate individual hydration requirements. This area of research, particularly regarding sodium losses, has been relatively understudied. We evaluated the sweat rate (SR), sweat sodium losses, and their relationship with body composition in professional soccer players in Cali, Colombia. *Materials and Methods*: Thirty-two male players, aged 24.3 (±5.2) years, from the Colombian main soccer league, underwent high-intensity training at 32 °C (with a relative humidity of 79%). The outcome variables included SR, calculated using weight loss and fluid intake; forearm sweat sodium concentration (FSCC), measured through the direct ion-selective electrode method; and estimated the predicted whole sweat sodium loss (PWSSL) in mmol. Predictor variables (body mass, fat, and muscle masses) were estimated using the Deborah Kerr anthropometry method. The association between predictors and outcomes was assessed using linear regression. *Results*: The mean FSCC, PWSSL, and SR were 26.7 ± 11.3 mmol/L, 43 ± 15.9 mmol/L, and 1.7 ± 0.5 L/h, respectively. Body mass positively predicted FSCC in unadjusted and age/fat-mass-adjusted models [Beta 1.28, 95% confidence interval (CI) 0.39–2.18, *p* = 0.006], and continued related to FSCC after adjustment for muscle mass with marginal significance [Beta 0.85, 95% CI −0.02 to 1.73, *p* = 0.056]. Muscle mass was associated with the PWSSL in unadjusted and age/fat-mass-adjusted models [Beta 2.42, 95% CI 0.58–4.26, *p* = 0.012] and sustained an association with marginal statistical significance after adjustment for body mass [Beta 1.86, 95% CI −0.35 to 4.09, *p* = 0.097]. *Conclusions*: Under hot tropical weather conditions, FSCC was relatively low among the players. Body mass was better associated with the FSSC, and muscle mass better related to the PWSSL. Body and muscle masses could be regarded as potential factors to be explored in the estimation of individual sodium needs. However, further studies are required to validate and contrast our findings.

## 1. Introduction

The primary ergogenic aid for athletes is hydration. A loss greater than 2% of body weight during exercise, linked to the sweat rate (SR), diminishes sports performance and compromises cognitive function [1]. However, hyperhydration without adequate electrolyte intake can lead to hyponatremia, which interferes with performance [2]. In some athletes, severe dehydration and/or hyponatremia can occur, disrupting sports performance and potentially leading to cardiovascular attacks, kidney damage, or other pathologies, and in extreme cases, even death [2].

According to Baker et al., despite existing hydration guidelines, fluid intake recommendations should be individualized, considering variables like environment, acclimatization, clothing, hydration status, and body composition [3,4,5]. Roughly 60% of the total mass is body water, but this varies depending on body composition [6]. Wang et al. established that in mammals (including humans), fat-free mass (FFM) correlates positively with body water content, indicating that approximately 75% of FFM contains water, while adipose tissue holds only about 10% [7]. Therefore, athletes with greater muscle mass might have higher fluid and electrolyte requirements.

In soccer players, moderate dehydration has been shown to reduce the covered distance skill by 13–15% [8]. Similarly, hydration with carbohydrate–electrolyte fluids in these athletes helps to maintain passing and shooting skills, as well as sprint performance [9]. Individual hydration requirements can be determined by measuring the sweat rate and sweat electrolyte concentration of each athlete [10]. Adjusting the sodium (Na+) intake during exercise promotes extracellular fluid retention [10]. Studies suggest that in cases of high sodium losses (>3–4 g Na), strategies should be employed to prevent disruptions in the hydroelectrolytic balance [11,12,13]. Additionally, while some studies have evaluated, and found a positive relationship between body mass and sweat rate [4,14], specific associations between body mass components like muscle and/or fat mass and fluid balance variables have not been thoroughly assessed. Understanding these associations could offer insights for refining hydration formulas to suit individualized needs.

Regarding environmental conditions, a few studies on fluid balance in athletes have shown higher SR and forearm sweat sodium concentrations (FSSCs) during the summer in countries with distinct seasons [2,10]. However, to the authors’ knowledge, there is limited information on SR and SSC in professional athletes training in tropical regions with constant hot weather and high humidity. Although tropical natives might be more predisposed to heat acclimatization (a lower core body temperature, reduced heart rate, and higher sweat rate), athletes performing in tropical hot weather might be more susceptible to exceeding a fluid deficit higher than 2% of their body weight (3–10%) [15].

Considering the above, we conducted a study to describe fluid balance variables and to assess the association between body composition (total, fat, and muscle masses) and SR and FSCC, and predicted the whole sweat sodium loss (PWSSL) in professional soccer players training in tropical weather conditions in the city of Cali, Colombia.

## 2. Materials and Methods

### 2.1. Study Population

The study adopted a cross-sectional design and employed a non-probabilistic purposive sampling method. A minimum sample size was determined based on the central limit theorem, assuming that a sample mean would follow a normal distribution with a sample size equal to or greater than 30. The research involved thirty-two male soccer players, aged 24.3 (±5.2) years, from Club Deportivo Cali, who were registered in the Colombian professional main soccer league (commonly known as the first division league) and were residents of Cali. Inclusion criteria encompassed adults (≥18 years old) registered in the professional main soccer league, with a normal medical assessment. Exclusion criteria included conditions that could impact physical performance or alter the fluid balance, such as injuries, rehabilitation, nutritional risks, or symptoms of infectious or gastrointestinal diseases within the week before the project tests. All participants provided informed consent, and the study received approval from the Ethics Committee of the Faculty of Health Sciences at Pontificia Universidad Javeriana Cali.

Soccer players underwent a medical assessment to confirm their healthy status, which included a resting 12-lead electrocardiogram (ECG). The study involved an anthropometrical evaluation to determine body composition, followed by a training session where drink intake was recorded, and forearm sweat sodium was measured.

### 2.2. Anthropometrical Evaluation and Body Composition Variables

This investigation focused on body mass (weight), fat, and muscle masses estimated using the Deborah Kerr anthropometry method (DK-ANT) [16]. Anthropometric measurements included weight (Tanita TB 300 A Scale, Tokyo, Japan), height (SECA stadiometer, Birmingham, UK), skinfold thickness (Harpenden CE, Herts, UK), circumferences (Rosscraff ORC tape measure, Surrey, CA, USA), and bone diameters (Rosscraff, Campbell caliper, Surrey, CA, USA). All measurements were conducted in a fasting state between 6:00 and 8:00 AM by an ISAK-level-III-credited nutritionist and dietitian following ISAK guidelines [17].

### 2.3. Fluid Balance Variables (SR, FSCC, and PWSSL)

Before conducting these estimations, all individuals urinated to empty their bladders of fluid content. The sweat rate (SR) was estimated using pre-training and post-training weights, the quantification of fluid intake during training in liters, and the training duration based on protocols described by Baker et al. and Goudet et al. [4,18]. These variables were incorporated into the following formula to calculate the sweat rate in liters per hour: ((initial weight (Kg) − final weight (Kg) + fluid intake (L) − urine volume (L))/hours of training). None of the soccer players urinated during the training session; hence, this value was not factored into the formula.

Sweat samples were collected using a standardized regional absorbent patch technique on the soccer players’ forearms. From these samples, the forearm sweat sodium concentration (FSSC) was determined using the direct ion-selective electrode method (DISE). The equipment employed included: Horiba Na-11 LAQUAtwin portable Na meter, 3M Tegaderm + non-adherent wound-dressing film, 20 mL syringes, 70% isopropyl alcohol, distilled water, gauze, and stainless steel forceps. For this procedure, the area was initially cleansed with alcohol and deionized water, following which the patch was affixed to the forearm. After the completion of the training session, the patch was removed. Sodium measurement was carried out using the portable Na meter, calibrated with a syringe to extract the sweat sample. The sodium value was recorded in PPM (parts per million) and converted from PPM to mmol/L. Predicted whole sweat sodium loss (PWSSL) in mmol was calculated using the equation by Baker et al. ([Forearm sodium × 0.57] + 11.05), and this value was multiplied by the estimated sweat volume [19]. Additionally, the PWSSL was converted from mmol to mg for descriptive purposes.

### 2.4. High-Intensity Training Characterization

The training session was the most intense weekly session scheduled during the competitive phase of the national tournament when a match was set. This session is regular in the soccer club and it was not designed by the researchers. Characterization of the training is based on data obtained from the global positioning system (GPS) by Catapult [20]. The GPS quantifies external load by measuring players’ distances covered and speeds attained, distinguishing between different intensities based on predefined thresholds. Typical thresholds for such movements include high-speed runs (>5.5 m/s) and running distances (>7.0 m/s) to differentiate them from lower-intensity movements. The training started at 9:00 a.m.

The training conditions were as follows: total distance covered (meters): 6588; Distance covered at a high intensity (meters): 406; sprint distance (meters): 79. The soccer players were in their usual microcycle or long week, signifying a scheduled match during the week. The training session comprised warm-up, central-phase, and cool-down segments, lasting for 100 min, of which 77 min were actively engaged, resulting in a total distance traveled of 6588 m.

The soccer players’ dietary regimen comprised a normocaloric diet with a high intake of protein, ranging from 1.3 to 1.7 g per kilogram of body weight. Carbohydrate intake fell within the range of 5 to 7 g per kilogram, with a contribution between 25 and 35% of fat. This regimen constituted the standard diet designed and provided by the soccer club, and no specific diet plan was formulated by the researchers.

### 2.5. Data Analyses

All analyses were conducted using Stata 14.0 software. The study variables were of a quantitative continuous nature. Their distributions were assessed graphically through histograms, demonstrating normal or near-normal distributions. Consequently, they were described in terms of mean and standard deviation. The fluid balance variables represent the average of all players in a single training session.

Pearson correlations were employed to test relationships between fluid balance parameters such as the weight loss %, drink intake, SR, FSSC, and PWSSL. The primary analysis of this study, which focused on evaluating the relationship between body mass, fat, and muscle masses (predictor variables) with the SR, FSSC, and PWSSL (outcome variables), utilized linear regressions. Beta regression coefficients were estimated as unadjusted, age-adjusted, and additionally adjusted for each specific body composition item to ascertain independent association patterns. For instance, regressions with fat mass as a predictor were adjusted separately for body mass and muscle mass. Collinearity among the body composition variables was assessed using the VIF command after each regression model. A *p*-value < 0.05 was considered statistically significant, while *p*-values falling within the range from 0.05 to 0.099 were deemed as trends or marginal statistical significance. We additionally estimated the effect size of the significant and marginally significant predictors on the outcome variables via post-regression calculation of omega-squared values. Field (2013) defined omega-squared ranges of how much variance in the outcome variables are accounted for by the predictor variables: <0.01—very small; 0.01 ≤ omega-squared < 0.06—small; 0.06 ≤ omega-squared < 0.14—medium; omega-squared ≥ 0.14—large [21].

## 3. Results

The environmental conditions in the city of Cali, Colombia, were 32 °C with a relative humidity of 79%. Table 1 outlines the general characteristics and body composition of the participants. The majority of the soccer players were forwards (40.6%). On average, the group was relatively young, with a mean age of 24.3 years, representing a stage of full physical capability in soccer. The mean values for fat and muscular masses were within the expected ranges.

With regard to fluid balance variables (Table 2), the volume of drink intake during training was lower than the sweat rate (mean 1.7 L/h). The FSSC was 26.7 ± 11.3 mmol/L. Six players (16.6%) did not lose weight or slightly gain due to hydration. Additionally, 40.6% of the footballers exhibited a body weight reduction of 1% or more, which could potentially impact their performance.

There were significant moderate-strength correlations observed among fluid balance variables (Table 3). The percentage of weight loss and volume of drink intake during training showed an inverse correlation, while these variables, in turn, positively correlated with the SR (Table 3). The FSSC did not exhibit a significant correlation with the SR.

There were no statistically significant relationships between body mass, fat mass, and muscle mass with SR, in both crude and adjusted models (Table 4). The regression coefficients were very close to zero, confirming that the changes in kilograms in the body composition markers were not associated with changes in SR units.

Body mass emerged as the most consistent positive predictor for the FSSC, displaying stronger and more statistically significant regression coefficients across unadjusted, age-adjusted, and fat/muscle-mass-adjusted models (Table 5).

In comparison to body mass and fat mass, muscle mass emerged as the strongest and most significant predictor of the PWSSL across various adjustment models (Table 6). The regression coefficients indicating the relationship between muscle mass and PWSLL (beta-coefficients ranged from 1.86 to 2.54) (Table 6) were stronger than those observed between the body mass and FSSC (beta-coefficients ranged from 0.85 to 1.28) (Table 5).

The fat mass showed no association with the FSSC or PWSSL in both unadjusted and adjusted models.

There were no signs of collinearity observed in the multivariable regression models described above.

The analysis of effect size revealed large effects (omega-squared value > 0.14) of the body mass on the FSSC (omega-squared = 0.20) and of the muscle mass on the PWSSL (omega-squared = 0.17) when both predictors were not simultaneously present in the multivariable model, adjusting for each other. The omega-squared values indicated a medium effect size on the outcomes in models containing both predictors, body and muscle masses (body mass effect size on the FSSC [omega-squared = 0.09]; muscle mass effect size on the PWSSL [omega-squared = 0.06]). This pattern aligns with the attenuated statistical significances observed for the body-mass–FSSC relationship (*p* = 0.056) when adjusted for muscle mass (Table 5), and for the muscle-mass–PWSSL relationship (*p* = 0.097) when adjusted for body mass (Table 6).

## 4. Discussion

This study evaluated, for the first time, the sweat rate and sweat sodium loss in professional soccer players from a tropical country and explored the associations of muscle and fat masses with the sweat sodium concentration. The forearm sweat sodium concentration (FSSC) was lower compared to previous reports in other sports and environmental conditions. The sweat rate (SR) did not exhibit any correlation with the FSSC. While body mass remained a consistent and positive predictor of the FSSC, muscle mass emerged as the strongest predictor of the predicted whole sweat sodium loss (PWSSL) in multivariable linear regression models.

The SR observed in the soccer players of this study is comparable to that reported in previous investigations, ranging from 1.1 L/h to 2.2 L/h [10,22]. However, the mean FSS was lower than reported in a few studies examining this sweat electrolyte variable. Baker et al. reported mean forearm sweat sodium concentrations ranging between 36 and 46 mmol/L in competitive/professional athletes across sports such as American football, baseball (36 ± 18 mmol/L), basketball, tennis, and soccer, noting no significant differences across these sports groups [4]. In their study, adult athletes exhibited significantly higher FSSCs compared to youths (47.0 ± 18.1 vs. 34.7 ± 15.4), and sex was not identified as a significant predictor of the FSSC.

Consistent with Baker et al., Yeargin et al. reported a higher mean FSSC in non-professional high school American football players aged 16–17 years compared to younger adolescents aged 13–15 years (40.4 ± 19.0 mmol/L and 27.3 ± 17.2 mmol/L, respectively) [23]. Conversely, Godek et al. described a much higher sweat sodium concentration in male American football players than Baker et al.’s study, with a mean of around 70 mmol/L across different field position groups [14]. It is worth noting that the mean temperature in Baker et al.’s study was 3 °C higher compared to Godek et al.’s study (28 °C vs. 25 °C) [4,14]. Recently, Surapongchai et al. reported the predicted whole sweat sodium loss (mmol) in tropical native recreational runners, although the data on the forearm sweat sodium concentration (FSSC), which was measured for the prediction, were not provided [15]. The Surapongchai et al. study is one of the very few investigations conducted in a tropical environment, and the predicted whole sodium loss in their runners exceeds the PWSSL found in the soccer players in our study (54 ± 27 vs. 43 ± 15.9 mmol) [15]. Discrepancies in temperature and humidity conditions, which were higher in our study (32 °C/79% vs. 29 °C/70%), might have influenced the disparity in the PWSSL. However, the two studies may not be methodologically comparable, since, in the Surapongchai et al. study, the intensity of running varied among the participants, and the distance and time covered by runners were relatively variable. Additionally, the expertise level of the runners was recreational, while the soccer players in our study were professional athletes competing at a high level. Surapongchai et al. concluded by emphasizing the need for research focused on competitive professional runners in tropical environments [15].

It is important to highlight that few studies on athletes have evaluated the FSSC or PWSSL with variations in their methodologies [10,22]. In our study, the FSSC was measured using the direct ion-selective electrode method (DISE), which has shown a good correlation with the reference method of ion chromatography (IChr) [10,18]. DISE is an accepted method for field research, since measurements can be promptly conducted upon sample collection, potentially avoiding technical biases due to transportation and storage if proper control is unattainable [10]. Despite the disparity in FSSC values, both Godek et al. and Yeargin et al. used the flame photometry method for their measurements [14,23], whereas Baker et al. used IChr [4]. The main methodological caution pertains to the use of the ion conductivity method, which measures electrolytes but is not an ion-specific technique, and thus not recommendable for the FSSC [10]. A methodological comparison by Goulet et al. encompassing sodium measurement methods (IChr, flame photometry, DISE, indirect ion-selective electrode, and ion conductivity) demonstrated that, in comparison to IChr, DISE was the most precise method (variation coefficient: 3.9%) [18]. Nonetheless, it has been suggested that results from the different methods should not be considered interchangeable [11].

The lower FSSC observed in the soccer players from our study could be attributed to individual adaptations to training and/or acclimatization, which might involve increased stroke volume, sweat dilution, and particularly solute reabsorption in high temperatures. Soccer players in Colombia are consistently exposed to high temperatures throughout the year due to the absence of temperate seasons in tropical countries. Specifically, the soccer players in our study train in Cali, a city characterized by hot weather with daytime temperatures ranging from 28 to 32 °C all year round and an average relative humidity of 97%.

The correlations among fluid balance variables seem to display variations in their strength and statistical significance across different studies. As anticipated, we observed that a higher percentage of weight loss corresponded to a greater volume of fluid intake. We discovered statistically significant moderate positive correlations between the sweat rate and drink intake, as well as between the sweat rate and the percentage of weight loss. Regarding the correlation between the sweat rate and drink intake, our findings align with a study by Godek et al., who reported a strong correlation approaching statistical significance (r = 0.57, *p* = 0.053) in American football athletes [14]. Similar to our study, Godek et al. estimated the sweat rate by calculating the change in mass adjusted for all fluids consumed between pre-practice and post-practice measurements of mass. However, Shirreffs et al. estimated sweat rates through a direct multiple-path gravimetric method, and did not find a significant relationship between the volumes of sweat loss and drink intake during training in 26 professional soccer players [24]. Due to the disparity in the findings and methods used for sweat rate estimation, significant correlations between the sweat rate and volume of fluid intake might be erroneous if the fluid intake is part of the calculation of the sweat rate. However, it is worth noting that the volume of fluid intake is not used as a multiplying factor but rather as an additional variable in the calculation formula, making it challenging to predict to what extent a correlation with the sweat rate might be redundant. Hence, studies comparing sweat rate estimations from diverse methods concerning fluid balance variables are warranted.

Meanwhile, in our study, there was no significant correlation between the sweat rate (SR) and the forearm sweat sodium concentration (FSSC) in soccer players. Previously, Baker et al. described a significant SR-FSSC relationship (beta coefficient = 3.34, *p* = 0.002) among multiple sports athletes [4], while Yeargin et al. reported a trend of a strong correlation between these two variables (r = 0.98, *p* = 0.077) in American football players [23]. However, our negative finding is in line with a study conducted on female soccer players (n = 13) by Kilding et al., which did not find a relationship between the SR and mean sweat sodium concentration (taken from different body regions) [25]. Kilding et al. sustained their negative finding based on small concentrations of electrolytes excreted in sweat. Kilding et al. attributed their negative finding to the small concentrations of electrolytes excreted in sweat. However, the mean sweat sodium concentration in their soccer players falls within the range reported in previous studies and tends to be relatively high (two measurements: 43.9 ± 15.0 and 46.2 ± 7.9 mmol/L) [25]. Considering the relatively lower FSSC in our study, it is plausible that with a smaller and narrower range of FSSCs, a correlation with the SR may not become evident. This lack of correlation might reflect actual mechanisms that are yet to be disclosed, which could also be population- or environment-dependent.

There is limited information available on the evaluation of the association between variables of body composition, and most of the few studies in this regard have focused primarily on body mass. Our finding of no association between the sweat rate (SR) and body mass contradicts studies by Godek et al. (2008) and Baker et al. (2016), which discovered positive relationships in American football players and athletes from other sports [4,14]. However, the strength of the relationship in these two studies notably differed, presumably due to differences in the adjustment approaches. In Godek et al.’s study with twelve male American football players, an unadjusted analysis showed a Pearson coefficient of 0.56 (*p* = 0.046). On the other hand, Baker et al. described a statistically significant yet very weak beta regression coefficient (0.006, *p* < 0.001) in a multivariable regression model that included age group, relative humidity, sex, and athlete level (recreational/competitive/professional) among 506 athletes from various sports [4,14].

Regarding the sweat sodium, both the body mass and muscle mass showed association patterns with these outcomes, with the body mass displaying a stronger relation to the forearm sweat sodium concentration (FSSC), and the muscle mass being more closely associated with the predicted whole sweat sodium loss (PWSSL). The reasons for this differential pattern are not clearly explained. Future studies could compare the relationship between body mass and muscle mass with the FSSC across different skin regions to clarify the specificity of our association patterns. In terms of potential physiological mechanisms, the finding that a greater muscle mass is associated with an increased whole sweat sodium loss might involve muscular adaptations occurring due to chronic exercise. These adaptations can include an increase in the size of muscle fibers, changes in contractile protein isoforms, a rise in the number of mitochondria depending on the type of training, an increase in the number of capillaries, and subsequently, enhanced blood flow to the active muscles. Additionally, this could lead to an increase in the supply of electrolytes such as sodium ions to these tissues [26,27,28]. Electrolytes such as sodium and potassium ions play pivotal roles in the function of both excitable and non-excitable cells. In muscle cells, these ions facilitate temporary alterations in the membrane potential, which instigate muscle contractions. However, the loss of sodium and water has been linked to muscle fatigue [29,30]. Consequently, heightened muscular activity results in an increased influx of sodium ions into these cells, followed by their efflux from muscle cells facilitated by the sodium–potassium ATPase pump. Ultimately, sodium ions are excreted in sweat through sweat glands [31]. Adaptations to the thermoregulation of sweat glands, particularly eccrine sweat glands, facilitate the excretion of sodium, primarily mediated by acetylcholine and, to a lesser extent, adrenaline [32,33,34]. These adaptations likely account for the findings observed in this study.

### Limitations and Strengths

Several limitations need to be acknowledged. Firstly, our study utilized a body composition methodology reliant on prediction equations derived from anthropometric measures, known as the Deborah Kerr anthropometry method (DK-ANT) [16]. The gold standard for estimating body composition is dual-energy X-ray absorptiometry (DXA) [35]. While a good correlation exists between these methods, DK-ANT may potentially overestimate the muscle mass [35]. Nevertheless, any overestimation, if present, is unlikely to bias the relationships observed between the body composition and sweat rate variables, as discrepancies between methods tend to be systematic in nature. Secondly, our study did not encompass evaluations of other groups of soccer players under varying weather and humidity conditions. Colombia, being a tropical country, exhibits diverse thermal environments across its regions. Future extensive studies could delve into differences in the sweat rate, FSSC, and PWSSL, and the relationships between body composition and fluid balance among athletes across regions in countries with thermal diversity. Thirdly, our study employed a cross-sectional design, limiting our ability to capture potential physiological adaptations in soccer players over time preceding our analyses. As such, our findings may not be broadly applicable to other populations.

One of the strengths of the present study is its approach comprising the simultaneous evaluation of body mass, and fat and muscle masses, to predict the sweat rate and sodium losses using a multivariable analysis. Additionally, it might be one of the first investigations exploring these connections and describing fluid balance variables among professional soccer players from a tropical Latin American country. These findings are expected to be used for future systematic reviews and meta-analyses in this field. Our results contribute to enhance the understanding of fluid and sodium losses in athletes based on body composition. Body and muscle masses could be examined as variables in equations for calculating sodium needs. However, it is important to interpret our findings cautiously until further validation is achieved through future studies employing similar methodological approaches.

## 5. Conclusions

The FSSC was relatively low in these male soccer players who trained in hot tropical weather conditions. Body mass had a better association with the FSSC, while muscle mass showed a better relationship with the PWSSL. Body and muscle masses could be regarded as potential factors to be explored in the estimation of specific sodium hydration needs. However, further studies in other populations of soccer players and also across various sports and diverse temperature/humidity conditions are required to validate and contrast our findings.

## Figures and Tables

**Table 1:** General characteristics and body composition of the study population.

Player Position	*n* (%)
Goalkeeper	4 (12.5)
Defender	8 (25)
Midfielder	7 (21.9)
Forward	13 (40.6)
	Mean ± SD
Age (years)	24.3 ± 5.2
Height (cm)	179.6 ± 5.5
Body mass (weight Kg)	76.3 ± 5.3
Fat mass (Kg)	15.7 ± 2.3
Muscle mass (Kg)	38.7 ± 2.3
Fat mass Kg/Kg of body weight	0.20 ± 0.02
Muscle mass Kg/Kg of body weight	0.50 ± 0.03
Training duration (min)	97.1 ± 5.9

**Table 2:** Fluid balance variables.

	Mean ± SD
Drink intake (L/h)	1.1 ± 0.4
Sweat rate (L/h)	1.7 ± 0.5
Forearm sweat sodium (mmol/L)	26.7 ± 11.3
Predicted whole sodium loss (mmol)	43 ± 15.9
Predicted whole sodium loss (mg))	0.9 ± 0.3
Percentage of body weight reduction after training	0.69 ± 0.63
Body weight reduction ≥1% *n* (%)	13 (40.6)

**Table 3:** Pearson correlations between fluid balance variables.

	% Weight Loss		Volume of Drink Intake (L)		Sweat Rate (L/h)		Forearm Sweat Sodium (mmol/L)	
	r	*p* Value	r	*p* Value	r	*p* Value	r	*p* Value
% weight loss			−0.431	0.014	0.574	0.001	−0.178	0.331
Volume of drink intake (L)					0.485	0.005	−0.065	0.724
Sweat rate (L/h)							−0.185	0.312
Forearm sweat sodium (mmol/L)								

**Table 4:** Linear regressions with the sweat rate (L/h) as outcome and the body mass and muscle and fat masses as predictors.

	Unadjusted	Adjusted for Age	Adjusted for Age and Body Mass	Adjusted for Age and Fat Mass	Adjusted for Age and Muscle Mass
	βeta (95% CI)	βeta (95% CI)	βeta (95% CI)	βeta (95% CI)	βeta (95% CI)
Body mass	−0.004 (−0.03 to 0.03)	0.004 (−0.03 to 0.04)	*	0.005 (−0.03 to 0.05)	−0.005 (−0.05 to 0.04)
	*p* = 0.795	*p* = 0.818		*p* = 0.793	*p* = 0.816
Fat mass (Kg)	−0.01 −0.09 to 0.06	0.0006 −0.082 to 0.084	−0.06 (−0.11 to 0.09)	*	−0.003 −0.089 to 0.081
	*p* = 0.695	*p* = 0.987	*p* = 0.892		*p* = 0.928
Muscle mass (Kg)	0.01 −0.04 to 0.07	0.02 −0.03 to 0.08	0.02 (−0.05 to 0.10)	−0.02 −0.04 to 0.08	*
	*p* = 0.654	*p* = 0.470	*p* = 0.477	*p* = 0.475	

* The beta-coefficient was not estimated because the predictor variable was the same covariate for adjustment. CI: confidence interval.

**Table 5:** Linear regression analysis with the forearm sweat sodium concentration (mmol/L) as the outcome and the total, fat, and muscle masses as predictors.

	Unadjusted	Adjusted for Age	Adjusted for Age and Body Mass	Adjusted for Age and Fat Mass	Adjusted for Age and Muscle Mass
	βeta (95% CI)	βeta (95% CI)	βeta (95% CI)	βeta (95% CI)	βeta (95% CI)
Body mass	**1.17** **(0.50 to 1.83)**	**1.22** **(0.49 to 1.95)**	*	**1.28** **(0.39 to 2.18)**	**0.85** **(−0.02 to 1.73)**
	***p =* 0.001**	***p =* 0.002**		***p =* 0.006**	***p =* 0.056**
Fat mass (Kg)	1.06 −1.46 to 3.60	1.51 1.15 to 4.18	−0.25 (−2.26 to 1.76)	*	1.04 −1.41 to 3.49
	*p =* 0.397	*p =* 0.255	*p =* 0.801		*p =* 0.392
Muscle mass (Kg)	**2.31** **0.51 to 4.10**	**2.54** **0.73 to 4.35**	1.06 (−0.41 to 2.54)	**2.42** **0.58 to 4.26**	*
	***p =* 0.014**	***p =* 0.008**	*p =* 0.153	***p =* 0.012**	

* The beta-coefficient was not estimated because the predictor variable was the same covariate for adjustment. Significant (*p* < 0.05) and marginally significant associations (*p* < 0.1 and >0.05) are shown in bold. CI: confidence interval.

**Table 6:** Linear regression analysis for the predicted whole sweat sodium loss (mmol) as the outcome and the total, fat, and muscle masses as predictors.

	Unadjusted	Adjusted for Age	Adjusted for Age and Body Mass	Adjusted for Age and Fat Mass	Adjusted for Age and Muscle Mass
	βeta (95% CI)	βeta (95% CI)	βeta (95% CI)	βeta (95% CI)	βeta (95% CI)
Body mass	**1.33** **(−0.05 to 2.06)**	**1.33** **(0.22 to 2.43)**	*	**1.39** **(0.02 to 2.70)**	0.68 (−0.63 to 2.00)
	***p =* 0.020**	***p =* 0.020**		***p =* 0.046**	*p =* 0.298
Fat mass (Kg)	1.06 (−1.46 to 3.68)	1.51 (−1.15 to 4.18)	−0.26 (−3.33 to 2.81)	*	1.04 (−1.41 to 3.49)
	*p =* 0.397	*p =* 0.255	*p =* 0.863		*p =* 0.392
Muscle mass (Kg)	**2.30** **(0.50 to 4.10)**	**2.54** **(0.73 to 4.35)**	**1.86** **(−0.35 to 4.09)**	**2.42** **(0.58 to 4.26)**	*
	***p =* 0.014**	***p =* 0.008**	***p =* 0.097**	***p =* 0.012**	

* The beta-coefficient was not estimated because the predictor variable was the same covariate for adjustment. Significant (*p* < 0.05) and marginally significant associations (*p* < 0.1 and >0.05) are shown in bold. CI: confidence interval.

## Data Availability

The data presented in this study are available on request from the corresponding author. The data are not publicly available due to privacy or ethical restrictions.
